# Association between SARS-CoV-2 Seroprevalence in Nursing Home Staff and Resident COVID-19 Cases and Mortality: A Cross-Sectional Study

**DOI:** 10.3390/v14010043

**Published:** 2021-12-28

**Authors:** Ania Wisniak, Lakshmi Krishna Menon, Roxane Dumont, Nick Pullen, Simon Regard, Richard Dubos, María-Eugenia Zaballa, Hélène Baysson, Delphine Courvoisier, Laurent Kaiser, Didier Pittet, Andrew S. Azman, Silvia Stringhini, Idris Guessous, Jean-François Balavoine, Omar Kherad, The SEROCoV-WORK + Study Group

**Affiliations:** 1 Unit of Population Epidemiology, Division of Primary Care Medicine, Geneva University Hospitals, 1211 Geneva, Switzerland; lakshmi.menon@etu.unige.ch (L.K.M.); roxane.dumont@hcuge.ch (R.D.); nicholas.pullen@hcuge.ch (N.P.); richard.dubos@hcuge.ch (R.D.); mariaeugenia.zaballa@hcuge.ch (M.-E.Z.); helene.baysson@unige.ch (H.B.); azman@jhu.edu (A.S.A.); silvia.stringhini@hcuge.ch (S.S.); 2 Institute of Global Health, Faculty of Medicine, University of Geneva, 1202 Geneva, Switzerland; 3 Department of Security, Population and Health, General Health Directorate, Canton of Geneva, 1211 Geneva, Switzerland; simon.regard@etat.ge.ch; 4 Division of Emergency Medicine, Geneva University Hospitals, 1211 Geneva, Switzerland; 5 Department of Health and Community Medicine, Faculty of Medicine, University of Geneva, 1211 Geneva, Switzerland; idris.guessous@hcuge.ch; 6 Care Quality Division, Medical and Quality Directorate, Geneva University Hospitals, 1211 Geneva, Switzerland; delphine.courvoisier@hcuge.ch; 7 Division of Laboratory Medicine, Geneva University Hospitals, 1211 Geneva, Switzerland; Laurent.Kaiser@hcuge.ch; 8 Geneva Center for Emerging Viral Diseases and Laboratory Virology, Geneva University Hospitals, 1211 Geneva, Switzerland; 9 Department of Medicine, Faculty of Medicine, University of Geneva, 1211 Geneva, Switzerland; didier.pittet@hcuge.ch (D.P.); jean-francois.balavoine@unige.ch (J.-F.B.); omar.kherad@latour.ch (O.K.); 10 World Health Organization Collaborating Center on Patient Safety, Infection Control Program, Department of Internal Medicine, Geneva University Hospitals, 1211 Geneva, Switzerland; 11 Department of Epidemiology, Johns Hopkins Bloomberg School of Public Health, Baltimore, MD 21205, USA; 12 University Center for General Medicine and Public Health, University of Lausanne, 1011 Lausanne, Switzerland; 13 Division of Primary Care Medicine, Geneva University Hospitals, 1211 Geneva, Switzerland; 14 Division of Internal Medicine, Hôpital de la Tour, 1217 Meyrin, Switzerland

**Keywords:** COVID-19, SARS-CoV-2, nursing homes, viral spread, transmission, seroprevalence

## Abstract

The burden of COVID-19 has disproportionately impacted the elderly, who are at increased risk of severe disease, hospitalization, and death. This cross-sectional study aimed to assess the association between SARS-CoV-2 seroprevalence among nursing home staff, and cumulative incidence rates of COVID-19 cases, hospitalizations, and deaths among residents. Staff seroprevalence was estimated within the SEROCoV-WORK+ study between May and September 2020 across 29 nursing homes in Geneva, Switzerland. Data on nursing home residents were obtained from the canton of Geneva for the period between March and August 2020. Associations were assessed using Spearman’s correlation coefficient and quasi-Poisson regression models. Overall, seroprevalence among staff ranged between 0 and 31.4%, with a median of 8.3%. A positive association was found between staff seroprevalence and resident cumulative incidence of COVID-19 cases (correlation coefficient R = 0.72, 95%CI 0.45–0.87; incidence rate ratio [IRR] = 1.10, 95%CI 1.07–1.17), hospitalizations (R = 0.59, 95%CI 0.25–0.80; IRR = 1.09, 95%CI 1.05–1.13), and deaths (R = 0.71, 95%CI 0.44–0.86; IRR = 1.12, 95%CI 1.07–1.18). Our results suggest that SARS-CoV-2 transmission between staff and residents may contribute to the spread of the virus within nursing homes. Awareness among nursing home professionals of their likely role in the spread of SARS-CoV-2 has the potential to increase vaccination coverage and prevent unnecessary deaths due to COVID-19.

## 1. Introduction

Since the beginning of the pandemic, the burden of COVID-19 has been greater among the elderly and people with comorbidities, who are at increased risk of severe disease, hospitalization, and death [1]. In particular, individuals living in nursing homes have been disproportionately afflicted worldwide, with a report across 22 countries showing that nursing home residents accounted for an average of 41% of all COVID-19-related deaths, despite representing only 0.73% of the general population [2]. In Switzerland, 49% of all COVID-19-related deaths occurred in nursing homes, not taking into account residents who died in hospitals [3].

Among the reasons behind the devastating impact of the pandemic on nursing homes, studies have suggested possible transmission of SARS-CoV-2 between personnel and residents of nursing homes [4,5] and other long-term care facilities [6], including occurrences of COVID-19 clusters in nursing homes likely initiated by staff members [7]. Importantly, a large proportion of asymptomatic or undetected COVID-19 cases among nursing home staff has been described in several studies worldwide and may contribute to silent SARS-CoV-2 transmission within nursing homes [8,9]. Of course, the higher vulnerability of this population to severe disease due to their advanced age is another important factor that should be considered. To what extent these different elements contribute to the high COVID-19 mortality within nursing homes remains unclear.

A study conducted in the canton of Geneva, Switzerland, after the first pandemic wave revealed that seroprevalence of anti-SARS-CoV-2 antibodies among essential workers varied widely across nursing homes, ranging between 0% of staff having developed antibodies to 31.4% [10], compared to a 7.8% (95% Credible Interval 6.8–8.9) seroprevalence in the general population during a similar time period [11]. The number of deaths has also varied widely across institutions, and it remains unknown whether the seroprevalence among staff, an indication of the circulation of the virus among employees, is correlated with the number of COVID-19 cases, hospitalizations, and deaths in nursing home residents. This information is of paramount importance at a time when vaccination hesitancy is becoming increasingly clear among healthcare and nursing home personnel at an international level, with vaccination rates as low as 42% in some high-income countries [12,13,14].

We aimed to assess the association between SARS-CoV-2 seroprevalence rates among staff and the cumulative incidence rates of COVID-19 cases, hospitalizations, and deaths among residents in nursing homes of the canton of Geneva, Switzerland, during the first pandemic wave between March and August 2020.

## 2. Materials and Methods

This cross-sectional study combined data on nursing home staff and residents from two different sources. Workers’ seroprevalence data were retrieved from the SEROCoV-WORK+ study, which was conducted between May and September 2020 across 29 out of 55 nursing homes in the canton of Geneva. All employees of nursing homes, including those from non-health sectors, were invited to participate and had their blood drawn once during the study period for serological testing. The inclusion period for sample collection in each nursing home is presented in the supplements (supplementary material, Figure S1). Sociodemographic data on nursing home staff were also collected during the same visit using self-administered questionnaires. The SEROCoV-WORK+ study has been described in detail previously [10]. Data on PCR-confirmed COVID-19 cases, hospitalizations and deaths due to PCR-confirmed COVID-19 among residents of nursing homes in the canton of Geneva, as well as PCR-confirmed COVID-19 cases among nursing home staff, were obtained from the Department of Security, Population and Health of the canton of Geneva for the period between March and August 2020. Data on the number of beds and monthly occupation rate for each nursing home were also made available. Serological status was determined with an enzyme-linked immunosorbent assay (ELISA; Euroimmun, Lübeck, Schleswig-Holstein, Germany, #EI 2606–9601 G) using the manufacturer’s recommended cutoff for positivity (≥1.1).

Of note, during the selected period, most nursing homes in the canton of Geneva had suspended or strongly limited visitations, and SARS-CoV-2 polymerase chain reaction (PCR) testing was restricted to symptomatic cases among nursing home residents and staff [15]. The cantonal federation of nursing homes in Geneva issued recommendations for safety and preventive measures to be implemented in nursing homes, including limitation of visitations to a strict minimum, social distancing when possible, hand sanitizing, wearing face masks in presence of the residents, and limited access to common spaces. However, there was no control of these measures and it is not known precisely to what extent individual nursing homes followed these recommendations. Further, many nursing home residents with advanced age and multiple comorbidities were not systematically hospitalized for severe COVID-19 cases in Switzerland, due to an unfavorable prognosis and the willingness to avoid excessive therapeutic measures, considering adequate non-invasive treatment can be provided within nursing homes.

Four nursing homes of the SEROCoV-WORK+ study were excluded from the analysis due to low numbers of participating staff (<10 participants) in the study. Twenty-five participants of the SEROCoV-WORK+ study working in more than one nursing home (out of 1071 total participants) were also excluded from the analysis to not interfere with results. Numerical data were summarized using medians and interquartile ranges. COVID-19 positivity, hospitalization, and death rates were calculated by dividing the total number of cases, respectively hospitalizations and deaths due to COVID-19, by the number of person-days in each nursing home for the period between March and June 2020, which was obtained by multiplying the number of beds by the occupation rate and the number of days within the selected period. The association between the proportion of seropositive staff by the nursing home and cumulative incidence of COVID-19 cases, hospitalizations, and deaths among residents were assessed using Spearman’s correlation coefficient and quasi-Poisson log-linear regression models. Within the regression models, we used the proportion of seropositive staff as the independent variable and the number of resident person-days at risk in each nursing home as an offset term. Results of the regression models are presented as incidence rate ratios (IRR) with their 95% confidence intervals. P-values lower than 0.05 were considered statistically significant. Statistical analysis was conducted using R statistical software (v. 4.0.3, R Foundation for Statistical Computing, Vienna, Vienna, Austria).

## 3. Results

A total of 25 nursing homes were included in the final analysis (Table 1). Overall, 33.8% of all employees of included nursing homes took part in the study, with a participation rate ranging between 16% and 61.3% of the total number of employees in individual nursing homes. The median number of participating workers by the nursing home was 39 (interquartile range (IQR) 20–50), ranging between 12 and 126 participants. Socio-demographic characteristics of nursing home workers included in the study are presented in the supplements (supplementary material, Table S1). Overall, 154 out of 1071 participating workers (14.4%) tested positive for anti-SARS-CoV-2 antibodies. Seroprevalence among staff ranged between 0 and 31.4% in individual nursing homes, with a median of 12.5% (IQR 4.4–20.5%). This contrasted with COVID-19 cases among staff reported by nursing homes, which ranged between 0 and 1.4% of total workers by nursing home, with only five (0.16%) COVID-19 cases detected among the staff of all included nursing homes for the study period. 

The median number of beds by nursing home was 80 (IQR 60–92) (range 48–235 beds) with a median occupation rate between March and June 2020 of 98.5% (IQR 97.2–99.6%). Overall, 16 out of 25 (64%) included nursing homes declared at least one COVID-19 case among their residents. All COVID-19 cases in residents were reported between March and May 2020 (supplementary material, Figure S2). Positive COVID-19 cases among residents ranged between 0 and 83 by nursing home, with a median of 5 cases (IQR 0–13); COVID-19-related hospitalizations ranged between 0 and 7, with a median of 0 hospitalizations (IQR 0–2); and deaths due to COVID-19 ranged between 0 and 33, with a median of 1 death per nursing home (IQR 0–4). The median cumulative incidence per 1000 person-days was 0.36 (IQR 0–0.69) for COVID-19 cases, 0 (IQR 0–0.10) for COVID-related hospitalizations, and 0.07 (IQR 0–0.19) for deaths due to COVID-19. 

**Figure 1:**
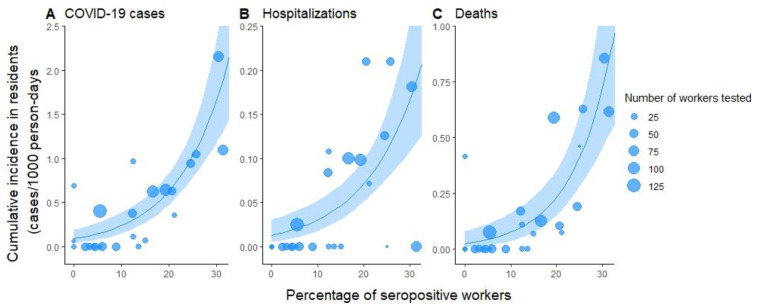
Percent of seropositive workers compared to the cumulative incidence of (**A**) PCR-confirmed COVID-19 cases, (**B**) COVID-related hospitalizations, and (**C**) deaths due to COVID-19 in nursing home residents. Each nursing home is represented by one point in the graphs. The size of dots is proportional to the number of workers with a serological test in each nursing home. Blue lines and envelopes represent quasi-Poisson regression model predictions and corresponding 95% confidence intervals.

We found a strong positive association between the proportion of seropositive staff in each nursing home and the cumulative incidence of COVID-19 cases among residents (Spearman’s correlation coefficient R = 0.72, *p* < 0.001), COVID-19-related hospitalizations (R = 0.58, *p* = 0.002) and deaths due to COVID-19 (R = 0.71, *p* < 0.001) (Figure 1 and Table 2). For each percent increase in seroprevalence in nursing home staff, we expect a 1.10-fold (95% CI 1.07–1.14) increase in confirmed COVID-19 cases in residents, a 1.09-fold (95% CI 1.05–1.13) increase in hospitalizations, and a 1.12-fold (95% CI 1.07–1.18) increase in the mortality rate among residents.

## 4. Discussion

In this study of 25 nursing homes in the canton of Geneva, Switzerland, we observed a significant positive association between SARS-CoV-2 seroprevalence rates among nursing home staff and cumulative incidence of COVID-19 cases, hospitalizations, and deaths among residents during the first pandemic wave. 

Our results are consistent with those of studies conducted in the United States [4] and Spain [5], which have shown an association between SARS-CoV-2 infection rates in nursing home staff and residents based on COVID-19 reported case counts and serological testing, respectively. Several studies have also identified SARS-CoV-2 infection rate within the community as an important predictor of infection rate in nursing homes [4,16]. However, our study shows that even within a small region such as the canton of Geneva, staff’s seroprevalence and resident infection rates vary widely between nursing homes, suggesting that other determinants of infectious spread are likely in play. 

Interestingly, we observed a large gap between the reported number of COVID-19 cases in nursing home staff and the seroprevalence rates identified in our study which were approximately 90-fold higher (0.16% vs. 14.4%). In comparison, the detection gap in the general population was only 10-fold higher at a similar time [17]. This suggests that many SARS-CoV-2 infections among staff members were likely asymptomatic or went unnoticed, therefore preventing the implementation of adequate isolation measures. Similar results have been found in a study in the United Kingdom, where 47% of care home staff were seropositive, while polymerase chain reaction (PCR) testing of nasal swabs came back negative for all individuals tested four weeks previously [9]. This is of great importance, as asymptomatic carriers may unknowingly contribute to the spread of SARS-CoV-2 within nursing homes [7,8].

The strengths of our study are the inclusion of nearly half of the nursing homes of the canton of Geneva and the availability of individual-level data on nursing home residents. Further, nursing homes are a particularly convenient setting to assess viral transmission, as residents tend to stay during prolonged periods of time, therefore limiting potential entry points of the virus through high patient turnover. However, our study comes with a number of limitations. First, the included nursing homes likely had different testing strategies at the time of the study, leading to potential underestimation of COVID-19 cases and related hospitalizations and deaths among residents, and limiting the generalizability of our results. Additionally, residents who died of COVID-19 in the hospital were not accounted for in the available data, therefore potentially further underestimating the number of deaths due to COVID-19. Regarding the periods of inclusion, there was some overlap in one nursing home between the diagnosed COVID-19 cases among residents and the serological testing among nursing home workers which occurred during the last week of May. However, considering that a large majority of resident cases in this nursing home were reported before May (supplementary material, Figure S2), it is unlikely that this overlap significantly affected the overall results. Further, participation rates of nursing home staff were relatively low. Finally, drawing firm conclusions from observational studies must be taken with caution as a significant association does not imply a causal relation, although SARS-CoV-2 transmission from staff to residents seems plausible given the restrictions around nursing home visitations at the time of the study in most facilities. We cannot ascertain that other determinants may have impacted the transmission in nursing homes. However, our results are supported by another study performed in a rehabilitation clinic of Geneva that combined genomic and epidemiological data into a Bayesian framework to model the directionality of transmission, where health care workers played an essential role in cross-transmission [6]. Further studies are needed to assess the role of different preventive and safety measures in the risk of nursing home staff-to-resident SARS-CoV-2 transmission.

## 5. Conclusions

Notwithstanding the inherent bias of cross-sectional studies, our results are consistent with the hypothesis that SARS-CoV-2 transmission occurs between staff and residents within nursing homes. This is important to acknowledge as vaccination rates among care home staff remain insufficient, despite evidence that vaccination leads to a decrease of viral spread and resident mortality in nursing homes [18]. Awareness among geriatric care professionals of their role in the spread of SARS-CoV-2 has the potential to increase vaccination coverage and prevent unnecessary deaths due to COVID-19. 

## Figures and Tables

**Table 1:** Characteristics of nursing homes, residents, and staff.

	Total Sample	Range by Nursing Home	Median (IQR) by Nursing Home
Nursing homes (N = 25)			
Number of beds	2317	48–235	80 (60–92)
Occupation rate (%)	96.9%	77.1–100%	98.6% (96.6–99.7)
Staff (N = 3167)			
Participating staff (n)	1071	12–126	39 (20–50)
Participation rate (%)	33.8%	16.0–61.3%	32.2% (20.8–45.9)
Seropositive staff (n)	154	0–22	3 (1–8)
Staff seroprevalence (%)	14.4%	0.0–31.4%	12.5% (4.4–20.5)
PCR-confirmed COVID-19 cases (n)	5	0–1	0 (0–0)
% staff with PCR-confirmed COVID-19	0.16%	0.0–1.4%	0.0% (0.0–0.0)
Residents			
PCR-confirmed COVID-19 cases (n)	229	0–83	5 (0–12)
Cumulative incidence of COVID-19 cases *	0.56	0.00–2.15	0.36 (0.00–0.69)
Number of COVID-19 hospitalizations	25	0–7	0 (0–2)
Cumulative incidence of hospitalizations	0.06	0.00–0.21	0.00 (0.00–0.10)
Number of COVID-19 deaths ^†^	89	0–33	1 (0–4)
Cumulative incidence of deaths *^†^	0.22	0.00–0.85	0.07 (0.00–0.19)

* per 1000 person-days. ^†^ COVID-19 deaths include only those occurring within the nursing homes, not in-hospital deaths. Abbreviations: SD = standard deviation, IQR = interquartile range, N = number.

**Table 2:** Correlation and quasi-Poisson log-linear regression results of the association between cumulative incidence per 1000 person-days of PCR-confirmed COVID-19 cases, COVID-19-related hospitalizations, and deaths due to COVID-19 among nursing home residents (outcome variables), and nursing home staff seropositivity (independent variable).

Outcome	Correlation	Quasi-Poisson Regression ^1^
	Spearman’s coefficient (95% CI)	Incidence rate ratio (95% CI)
COVID-19 cases	0.72 (0.45–0.87) *	1.10 (1.07–1.14) *
Hospitalizations	0.59 (0.25–0.80) **	1.09 (1.05–1.13) *
Deaths	0.71 (0.44–0.86) *	1.12 (1.07–1.18) *

Abbreviations: 95% CI = 95% confidence interval; PCR = polymerase chain reaction; * *p* < 0.001; ** *p* = 0.002. ^1^ Dispersion parameter taken to be 4.16.

## Data Availability

Study data that underlie the results reported in this article can be made available to the scientific community after deidentification of individual nursing homes and participants, and upon submission of a data request application to the investigator board via the corresponding author.

## Supplementary Materials

The following supporting information can be downloaded at: https://www.mdpi.com/article/10.3390/v14010043/s1, Figure S1: Inclusion period by nursing home for serological sampling among staff; Figure S2: Number of monthly COVID-19 cases among residents by nursing home; Table S1: Socio-demographic characteristics of nursing home staff.
