# Supplementary material for: Association between SARS-CoV-2 Seroprevalence in Nursing Home Staff and Resident COVID-19 Cases and Mortality: A Cross-Sectional Study
Source: Viruses. 2021 Dec 28;14(1):43. doi: 10.3390/v14010043 (PMC8779720; doi:10.3390/v14010043)
Supplement: Supplementary file 1 [file viruses-14-00043-s001.zip › viruses-1452003-supplementary.pdf]

Supplemental Material

Association between SARS-CoV-2 Seroprevalence in Nursing Home Staff and Resident COVID-19 Cases and Mortality: A Cross-Sectional Study

Ania Wisniak, Lakshmi Krishna Menon, Roxane Dumont, Nick Pullen, Simon Regard, Richard Dubos, María-Eugenia Zaballa, Hélène Baysson, Delphine Courvoisier, Laurent Kaiser, Didier Pittet, Andrew S. Azman, Silvia Stringhini, Idris Guessous, Jean-François Balavoine, Omar Kherad, and the SEROCoV-WORK + study group: Victoria Alber, Isabelle Arm-Vernez, Donatien Bachmann, Stéphanie Baggio, Gil Barbosa Monteiro, Hélène Baysson, Patrick Bleich, Isabelle Boissel, Prune Collombet, Philippine Couson, Alioucha Davidovic, Clement Deiri, Divina Del Rio, Carlos de Mestral, David De Ridder, Yaron Dibner, Paola D'ippolito, Joséphine Duc, Roxane Dumont, Isabella Eckerle, Nacira El Merjani, Gwennaelle Ferniot, Antoine Flahault, Natalie Francioli, Marion Frangville, Carine Garande, Laurent Gétaz, Pamela Giraldo, Fanny Golaz, Julie Guérin, Ludivine Haboury, Séverine Harnal, Victoria Javet, Amélie Laboulais, Gaëlle Lamour, Xavier Lefebvre, Pierre Lescuyer, Andrea Jutta Loizeau, Fanny-Blanche Lombard, Elsa Lorthe, Chantal Martinez, Kourosh Massiha, Ludovic Metral-Boffod, Benjamin Meyer, Khaled Mostaguir, Mayssam Nehme, Natacha Noël, Nicolas Oederlin, Francesco Pennacchio, Javier Perez-Saez, Dusan Petrovic, Attilio Picazio, Jane Portier, Géraldine Poulain, Caroline Pugin, Barinjaka Rakotomiaramanana, Zo Francia Randrianandrasana, Aude Richard, Viviane Richard, Sabina Rodriguez-Velazquez, Lilas Salzmann-Bellard, Leonard Thorens, Simon Torroni, David Vidonne, Guillemette Violot, Nicolas Vuilleumier, Zoé Waldmann, Manon Will, Sabine Yerly

---

Table S1. Socio-demographic characteristics of nursing home staff (N=1'071).

Category	N (%)
**Gender**	
Female	846 (79.0)
Male	225 (21.0)
**Age category**	
18-34	209 (19.5)
35-49	426 (39.8)
50-65	421 (40.2)
Other*	5 (0.5)
**Mean age ± SD**	45.1 ± 11.0
**Education level**	
No formal diploma	50 (4.7)
Primary education	174 (16.2)
Apprenticeship	265 (24.7)
Secondary education	230 (21.5)
Tertiary education	323 (30.2)
Other	29 (2.7)
**Occupation**	
Nurse / assistant nurse	490 (45.8)
Home help / personal assistance	40 (3.7)
Physicians	4 (0.4)
Other health care workers	118 (11.0)
Cleaning agent	125 (11.7)
Restaurant personnel	92 (8.6)
Administrative staff	84 (7.8)
Social worker	54 (5.0)
(Deputy) director	24 (2.2)
Other**	36 (3.4)
Missing	4 (0.4)

* Participants aged 17 (2), 70, 73 and 77 years old. ** Includes security agents/police officers (4), construction workers (6), cashiers/tellers (8), drivers (3), others (15).

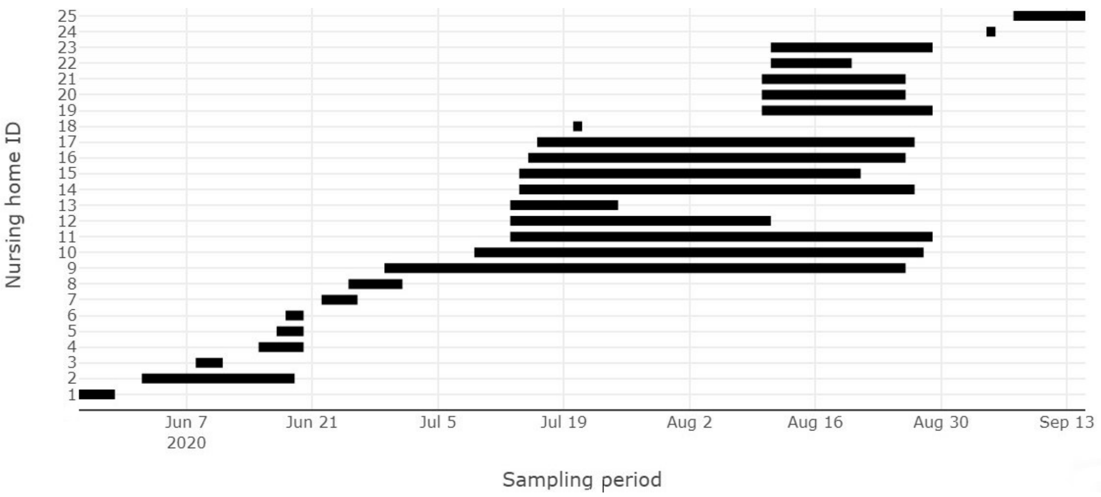

Figure S1. Inclusion period by nursing home for serological sampling among staff.

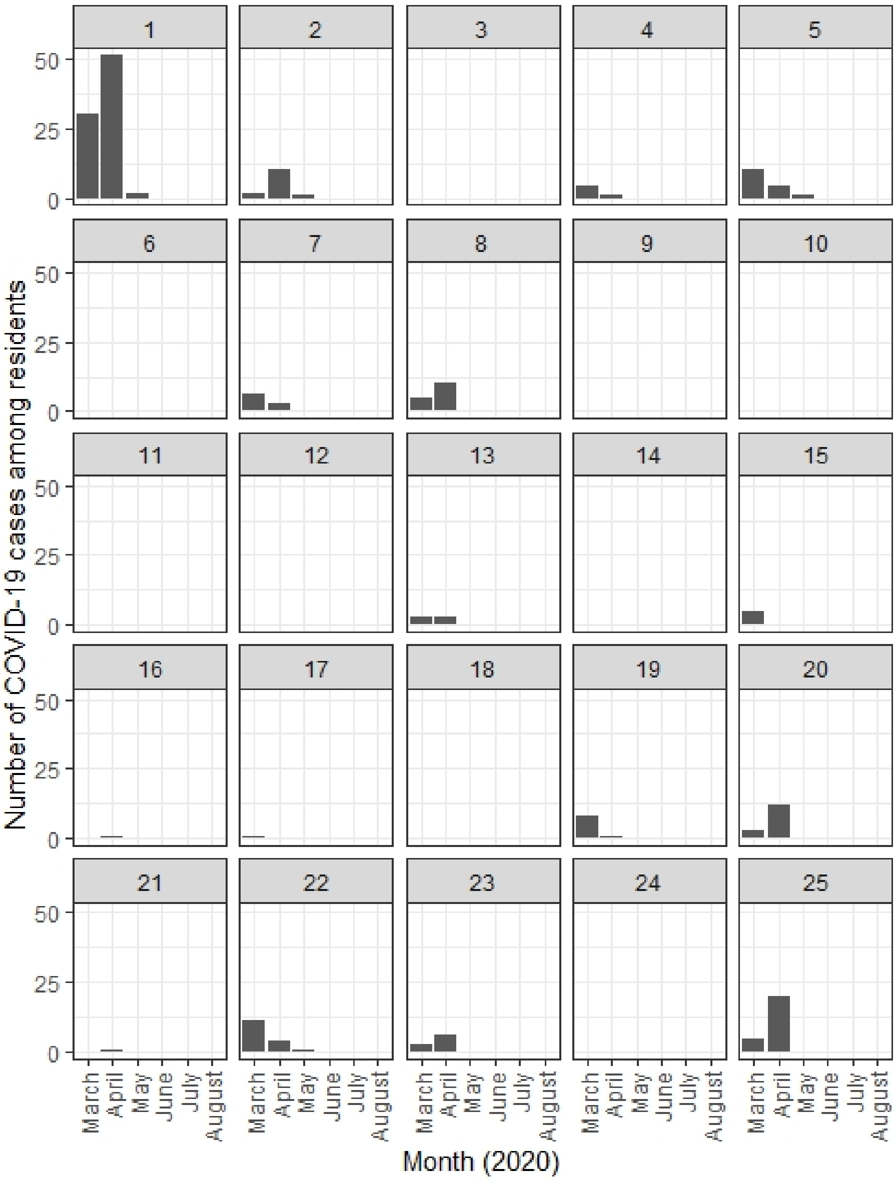

Figure S2. Number of monthly COVID-19 cases among residents by nursing home.
